# Silver Nanoparticles@Zeolite Composites: Preparation, Characterization and Antibacterial Properties

**DOI:** 10.3390/ma18173964

**Published:** 2025-08-24

**Authors:** Gospodinka Gicheva, Marinela Panayotova, Orlin Gemishev, Sergei A. Kulinich, Neli Mintcheva

**Affiliations:** 1 Department of Mineral Processing and Recycling, University of Mining and Geology, 1700 Sofia, Bulgaria; g.gicheva@mgu.bg (G.G.); marichim@mgu.bg (M.P.); 2 Faculty of Biology, University of Sofia ‘’St. Kliment Ohridski’’, 1000 Sofia, Bulgaria; o.gemishev@uni-sofia.bg; 3 Research Institute of Science and Technology, Tokai University, Hiratsuka 259-1292, Japan; skulinich@tokai-u.jp; 4 Department of Engineering Geoecology, University of Mining and Geology, 1700 Sofia, Bulgaria

**Keywords:** silver nanoparticles, clinoptilolite, Ag NPs@natural zeolite, nanocomposites, antibacterial properties

## Abstract

The presence of various Ag species (Ag^+^ ions, Ag clusters, and Ag nanoparticles (NPs)) in Ag-zeolite nanocomposites strongly influences their catalytic, photocatalytic, and antibacterial properties. To tailor materials for specific applications, it is essential to employ strategies that control the redox processes between Ag^+^ and Ag^0^ and facilitate the formation of active Ag-containing composites. In this study, we present a comparative analysis of Ag-zeolite nanocomposites, focusing on their synthesis methods, structural characteristics, and antibacterial activity against *Escherichia coli*. Ag NPs were synthesized using three approaches: solid-state thermal reduction, chemical reduction in aqueous solutions with a mild reducing agent (sodium citrate, Na_3_Cit), and chemical reduction with a strong reducing agent (sodium borohydride, NaBH_4_). The resulting materials were characterized by X-ray diffraction (XRD), diffuse reflectance UV–Vis spectroscopy (DR UV–Vis), X-ray photoelectron spectroscopy (XPS), and transmission electron microscopy (TEM), while antibacterial activity was assessed using biological assays. Microscopic and spectroscopic analyses confirmed the formation of Ag NPs and the co-existence of immobilized Ag^+^ ions within the zeolite framework. The specific influence of the treatment method of Ag^+^-zeolite on the presence of silver species in the nanocomposites and their role in antibacterial properties were evaluated. The highest antibacterial efficiency was observed in the nanocomposite produced by thermal treatment of Ag-exchanged zeolite. Thus, the crucial function of Ag^+^ ions in the mechanism of bacteria cell death was suggested.

## 1. Introduction

Silver nanoparticles (Ag NPs) have become an integral part in advanced nanotechnology due to their unique optical, electronic, and catalytic/photocatalytic properties [1,2,3,4,5,6,7,8]. A significant part of research on Ag NPs is dedicated to their well-established antibacterial properties and their incorporation into new functional materials with diverse applications in medicine [9], environmental science [10], and materials engineering [11], for example, in antibacterial textiles [12,13], wound bandages [14], active packages for food [15], and coatings [16], as well as materials used in water treatment [17,18]. Among many available supports, natural zeolites have been proven as an effective framework for the stabilization of silver species, promoting their uniform dispersion, size, and controlled release in solution [19,20].

Zeolites are microporous crystalline aluminosilicates, possessing well-defined pore structures, high surface areas, and ion-exchange capabilities that account for the accommodation of metal ions and nanoparticles [21,22]. The facile ion exchange process is widely used for incorporation of silver ions into the zeolite matrix, while subsequent chemical or thermal reduction transforms them into metallic nanoparticles, leading to the formation of hybrid materials with multifunctional properties [20].

Many methods for preparation of Ag-zeolite composites have been explored, including, most popularly, chemical reduction using reducing agents such as NaBH_4_ or citrate [23,24,25], formaldehyde, or saccharides (e.g., glucose, galactose, lactose, and maltose) [26]. While these reactions typically yield a high conversion of silver ions to metallic silver, the control of particle size and distribution remains challenging. Alternative approaches are photoreduction techniques [27] and thermal treatments [28] that produce highly crystalline, metallic silver nanoparticles with uniform size and thermal stability [29]. The choice of method significantly affects the size, distribution, and oxidation state of silver species, which in turn influence the activity and efficiency of composites in applications such as antimicrobial coatings, water purification systems, and active packaging.

In this study, we present the synthesis and characterization of Ag-containing zeolite composites using natural clinoptilolite as a support. Three different methods—thermal reduction and chemical reduction in solution by weak and strong reductants—were applied to produce materials with varying ratios of Ag^+^ ions and Ag NPs. The antibacterial activity of the resulting nanocomposites was evaluated against *Escherichia coli*, and structure–property relationships were investigated using spectroscopic and microscopic techniques and biological assays.

## 2. Materials and Methods

### 2.1. Materials

Silver nitrate (AgNO_3_), trisodium citrate (Na_3_Cit.2H_2_O), sodium tetrahydrido borate (III) (NaBH_4_) and triethanolamine (TEA; ≥98%) were purchased by Merck KGaA, Darmstadt, Germany. All reagents were used without any further purification. A natural zeolite from the region of East Rhodopes, Bulgaria, was used for all experiments, after proper washing and purification. The composition of the natural material is 76% clinoptilolite and 24% barrerite [30,31].

### 2.2. Preparation and Characterizations

#### 2.2.1. Preparation of Ag-Modified Zeolite (Ag^+^-Zeo)

As we previously reported, to increase the ion-exchange capacity of natural zeolite, it was modified by turning it into its sodium form, Na-form, by placing the zeolite in contact with 2 M NaCl solution at a solid-to-liquid ratio of 1:10, at room temperature for 7 days. The next step included immersing the Na-form zeolite in 0.1 M solution of AgNO_3_ at pH 6 and a solid-to-liquid ratio of 1:20 under stirring for 4 h to achieve silver ions loaded on zeolite. After filtration, washing, and drying, Ag-modified zeolite (denoted as Ag^+^-Zeo) was produced [31].

#### 2.2.2. Silver Nanoparticles@zeolite Composites

The silver loaded clinoptilolite, Ag^+^-Zeo, was used as a precursor for preparation of the studied samples. The Ag NPs@zeolite composites were formed by thermal reduction in the solid state, and chemical reduction in aqueous solutions with mild (Na_3_Cit) and strong (NaBH_4_) reducing agents, so that the samples Ag-Zeo-1, Ag-Zeo-2, and Ag-Zeo-3, respectively, were obtained. All samples were further examined by XRD, DR UV–Vis, TEM, and XPS methods for structural characterization as well as the antibacterial tests against *Escherichia coli* that were carried out.

##### Synthesis of Ag NPs@zeolite Composite by Thermal Reduction (Ag-Zeo-1)

One gram of the solid material, Ag^+^-Zeo, was placed in a porcelain crucible and heated at 400 °C for two hours in the presence of air and absence of light. After that, the crucible was cooled down to room temperature in a vacuum desiccator in a dark place. Interestingly, no visible change was observed after the thermal treatment and the material retained its initial beige color. The prepared Ag NPs@zeolite composite is denoted as Ag-Zeo-1.

##### Synthesis of Ag NPs@zeolite Composite by Chemical Reduction with Na_3_Cit (Ag-Zeo-2)

One gram of Ag^+^-Zeo zeolite was suspended in a 20 mL solution of sodium citrate with a concentration of 2 × 10^−3^ M under magnetic stirring at room temperature, and then 0.250 mL triethanolamine (TEA) was added to the reaction mixture under constant stirring. The reaction system was kept in the dark to avoid the photochemical reduction of silver ions to silver. After mixing for 4 h at room temperature, the suspension changed color from beige to dark brown. The fine powder was filtrated, washed three times with distilled water, and dried at room temperature in a dark place. The sample is denoted as Ag-Zeo-2.

##### Synthesis of Ag NPs@zeolite Composite by Chemical Reduction with NaBH_4_ (Ag-Zeo-3)

One gram of Ag^+^-Zeo was suspended in 40 mL of freshly prepared NaBH_4_ solution with a concentration of 5 × 10^−2^ M in an ice bath under magnetic stirring [24,32]. A vigorous reaction took place with the intense release of bubbles of hydrogen gas and immediate change of color from beige to black. The precipitate was left to settle down on the bottom of the container and then isolated from the mixture by filtration. It was washed three times with distilled water and kept drying at room temperature in a dark place. The sample is denoted as Ag-Zeo-3.

#### 2.2.3. Ag NPs@zeolite Composites’ Characterization

The nanomaterials’ composition, structure, and morphology were studied by X-ray diffraction analysis (XRD), transmission electron microscopy (TEM), diffuse reflectance UV–Vis spectroscopy (DR UV–Vis), and X-ray photoelectron spectroscopy (XPS). The XRD data were obtained on a Bruker D8 Advance powder diffractometer (Bruker, Karlsruhe, Germany) with Cu Kα radiation. The DR UV–Vis was used to demonstrate the formation of silver clusters and nanoparticles in zeolite based on their characteristic peaks in the range of 300–500 nm. The spectra were taken on an Evolution 300 UV–Vis Spectrophotometer (Thermo Fisher Scientific, Waltham, MA, USA) by using spectralon as a background. The Kubelka–Munk plots against wavelength were drawn and presented. TEM was applied to visualize the size and shape of the Ag NPs loaded on zeolite. The TEM images were observed on a JEOL JEM-2100 microscope (JEOL, Tokyo, Japan) with an accelerating voltage of 200 kV. To evaluate the oxidation state of silver in the zeolite, X-ray photoelectron spectroscopy was used. The measurements were carried out on a spectrometer Escalab-MkII (VG Scientific, East Grinstead, UK). The electrons were excited with AlKα radiation (hν = 1486.6 eV) at low power (5 mA/6 kV) of the X-ray source to minimize the effect of irradiation on the sample during data acquisition. The binding energy calibration was done by using the strongest O1s line in the spectra centered at 532.7 eV.

#### 2.2.4. Antibacterial Activity of Ag NPs@zeolite Composites

The evaluation of the nanocomposites’ antibacterial activity was performed against *Escherichia coli*. The control type strain, *Escherichia coli* 3398 for testing, was purchased by the National Industrial Bank for microorganisms and cell cultures in Bulgaria. Cultivation of the test microorganism, the preparation of peptone water, and the inoculation of *Escherichia coli* were performed using the standard laboratory procedure described previously [31].

To determine the minimum inhibitory concentration (MIC) and the degree of the bactericidal action of the synthesized Ag NPs@zeolite composites, a sample of the nanocomposite at a concentration range from 0.2 to 5.0 mg/mL was added to the inoculated (with *Escherichia coli* 3398) peptone water (PW). Samples were cultivated for 24 h at 37.0 ± 0.1 °C. Then, using 1 mL of the suspension, a series of consecutive dilutions were made in sterile distilled water (in three parallel samples). Aliquots of 0.1 mL from each dilution in sterile conditions were transferred in Petri dishes containing agarose into Luria medium (0.5 g/L NaCl). The inoculated Petri dishes were incubated at 37.0 ± 0.1 °C for 24 h. Bacterial colonies were counted, and the number of viable cells was reported as colony-forming units per mL (CFU/mL). Natural zeolite (added to PW at concentrations the same as the concentrations of the composite suspensions used) and *Escherichia coli*-inoculated peptone water (without any added zeolite and nanocomposites) were used as control samples. Controls were treated (dilutions and incubations) in the same way as the other samples. The antibacterial activity (%, percent) of each type of nanocomposite was calculated based on a comparison between the bacterial growth in its presence and in the control (suspension of *Escherichia coli* in PW without any zeolite), which after inoculation as described above showed a bacterial growth of 10^7^ CFU/mL.

## 3. Results and Discussion

Zeolites have stable Si−O−Al frameworks that accommodate metal cations (K^+^, Na^+^, Ca^2+^, and Mg^2+^), offering various interactions with other metal ions, such as ion exchange and the incorporation of ions into the channels and molecular-sized cavities, where under certain conditions, metal clusters can be formed. The dimensions of the openings, mobility of ions, and their environments provide a wide range of processes that may control the formation of metal species in zeolites. Thus, instead of considering zeolites as passive supports bearing ions and nanoparticles, we investigate the metal–zeolite interactions, as well as the physical and chemical processes taking place in the solid state and in the solution of Ag-modified zeolite.

Some authors immobilize the Ag^+^ ions on zeolite from AgNO_3_ and immediately add the reducing agent to get Ag NPs deposited on the zeolite surface [32,33]. Others reduce Ag^+^-loaded ions by heating the Ag–zeolite under H_2_ flow [34]. Ribeiro et al. separately produce Ag NPs in solution by naturally occurring reducing extracts and then mix the Ag NP suspension with zeolite to get nanocomposites [35].

In our previous study, we found that the loading of Ag^+^ ions onto Na-modified zeolite was greater than that onto natural and H-modified zeolites, resulting in Ag-richer zeolite. Therefore, in this study, a Na-modified zeolite was used as support for the immobilization of Ag^+^ ions by ion exchange from an aqueous AgNO_3_ solution [31]. The precursor silver-ion-loaded zeolite, Ag^+^-Zeo, was treated in three different ways to produce Ag NPs@zeolites: one part was heated at 400 °C for 2 h in air (sample Ag-Zeo-1), the second portion was reacted with sodium citrate in an aqueous solution at room temperature (sample Ag-Zeo-2), and the third one was added to an aqueous solution of sodium tetrahydrido borate under ice-cooling (sample Ag-Zeo-3).

A comparative study of Ag NPs@zeolite nanocomposites revealed that the structure, morphology, NP size and distribution, and antibacterial properties vary depending on the method of preparation.

**Figure 1:**
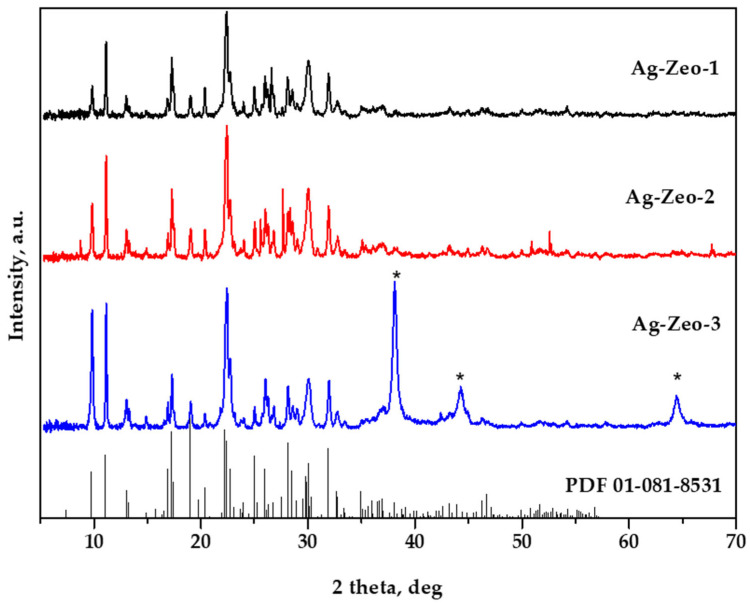
XRD patterns of samples Ag-Zeo-1, Ag-Zeo-2, and Ag-Zeo-3. XRD patterns of Ag-exchanged clinoptilolite (PDF 01-081-8531). XRD patterns of metallic silver (PDF 04-0783) at 2θ = 38.1, 44.3, and 64.4° are denoted with asterisks.

The XRD analysis of as-prepared samples revealed that the immobilization of Ag^+^ ions and their reduction to Ag clusters and nanoparticles have no perceptible effect on the zeolite structure and crystallinity (Figure 1). In our previous study, we demonstrated very similar XRD patterns of natural zeolite and Ag-loaded zeolite, indicating that the zeolite structure is not altered by the immobilization of silver ions [36]. In Figure 1, the XRD patterns of Ag-exchanged clinoptilolite (PDF 01-081-8531) are given as a reference. There are no signals for silver nanoparticles after heating the solid Ag^+^-Zeo and treating the Ag^+^-Zeo suspension with Na_3_Cit, for samples Ag-Zeo-1 and Ag-Zeo-2, respectively. This is not surprising, because the Ag NPs formed in these samples are very small and evenly distributed among the zeolite crystals, so that XRD patterns for the silver phase are not registered. Similar results were observed by other authors as well [37]. However, the XRD patterns of sample Ag-Zeo-3 contain peaks of metallic Ag at angular positions (2θ) 38.1°, 44.3°, and 64.4°, corresponding to the cubic phase of Ag formed by the reduction of silver ions with NaBH_4_ [33,35,38].

**Figure 2:**
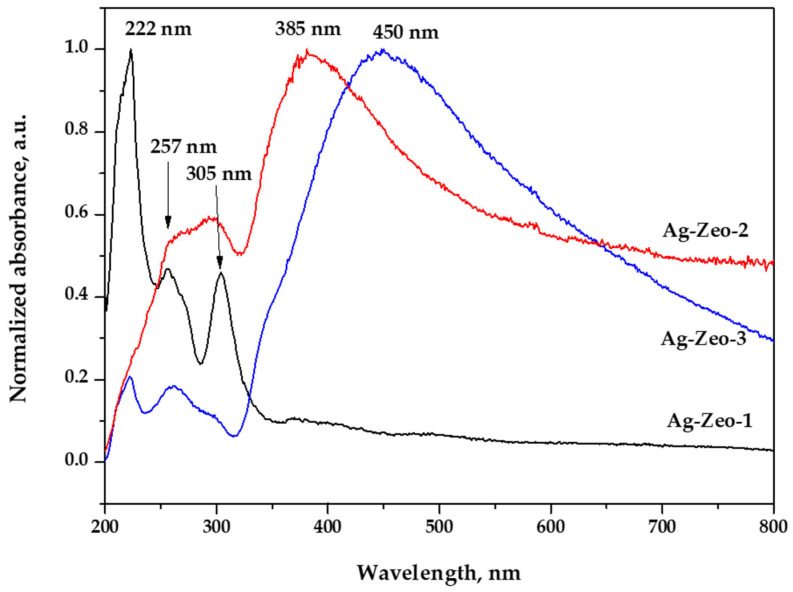
UV–Vis diffuse reflectance spectra of samples Ag-Zeo-1, Ag-Zeo-2, and Ag-Zeo-3.

The diffuse reflectance UV–Vis spectroscopy (DR UV–Vis) is often used to determine the presence of various silver species (Ag^+^, Ag clusters, and Ag NPs) in silver-containing zeolites. The DR UV–Vis spectra of the three samples are shown in Figure 2, where besides the typical zeolite peak at 257 nm, Ag NP peaks at 385 and 450 nm were also observed for the samples Ag-Zeo-2 and Ag-Zeo-3, respectively. The characteristic UV–Vis bands and their assignment are listed in Table 1.

The peak at 257 nm, that is present in all samples, is due to charge transfer from oxide ions, specifically O^2-^ to Al^3+^ ions located at specific sites in the zeolite framework [39,40].

In the Ag-Zeo-1 spectrum, the high intensity peak at 222 nm is attributed to the charge transfer band originating from an interaction between Ag^+^ ions and the oxygen atoms in the zeolite substrate, while the peak at 305 nm is due to Ag clusters, and the shoulder at 380 nm arises from small Ag NPs (see TEM data), both formed during the thermal reduction of Ag^+^-Zeo at 400 °C [37,39,41].

These results indicate that, during thermal treatment, immobilized Ag^+^ ions are slowly reduced to metallic silver, and then silver clusters and/or Ag NPs are formed. These processes are affected by the limited mobility of Ag^+^ ions in the solid state and by the spatial constraints caused by the narrow channels of the zeolite’s structure and intercrystallite moieties.

Based on quantum calculations of the geometry and electron transitions, Gurin et al. proposed the stabilization of Ag_8_^+^ clusters within the mordenite cavities, having first allowed transitions at wavelengths of 323 and 299 nm. They experimentally observed the peaks at 318–323 nm and 280–285 nm [42]. Other authors reported a peak at 310 nm for Ag_m_^0^ clusters in Ag-faujasite zeolite [43], and peaks at 310 and 325 nm for Ag-clinoptilolite [29].

In the spectra of the samples Ag-Zeo-2 and Ag-Zeo-3, the lower intensity of the peak at 222 nm related to the lower fraction of Ag^+^ ions in the zeolite network and higher degree of transformation of Ag^+^ ions to Ag^0^. Similarly, the band at 305 nm is weaker for Ag-Zeo-2 and very weak for Ag-Zeo-3, indicating the agglomeration of Ag clusters to larger particles, which is confirmed by the strong absorption bands at 385 nm and 450 nm, respectively. The latter are assigned to the surface plasmon resonance in Ag NPs and are characteristic bands for such particles. The higher wavelength shift and appearance of the maximum at 450 nm for Ag-Zeo-3 is associated with the formation of larger Ag NPs during NaBH_4_-assisted reduction in solution [37,43,44,45]. The broad absorption above 500 nm for both Ag-Zeo-2 and Ag-Zeo-3 arises from silver colloidal particles obtained in the solution during reduction by Na_3_Cit and NaBH_4_ and deposited onto the external surface of the zeolite.

When summarizing the data obtained from the DR UV–Vis spectra of the three composites Ag-Zeo-1, Ag-Zeo-2, and Ag-Zeo-3 (Table 1 and Figure 2), one can conclude that all materials demonstrate the characteristic bands of the zeolite substrate (222 nm and 257 nm) due to a stable zeolite framework and different levels of Ag^+^ ion content. The band at 305 nm shows that there are Ag clusters present in all composite materials regardless of the preparation method, but their aggregation is facilitated in solution that led to a well-pronounced band at higher wavelengths. For this reason, the spectra of chemically reduced Ag NPs show additional broad and intense bands at 385 nm and 450 nm, which are typical for Ag NPs, pointing to the conclusion that the initially formed Ag clusters continue to grow and form Ag NPs with a larger size in the Na_3_Cit and NaBH_4_ solutions. Thus, in the presence of a reductant, Ag^+^ ions in solution undergo fast reduction to metallic silver, followed by aggregation. The adsorbed Ag^+^ ions detach from the crystal surface and migrate into the solution. There, silver nanoparticles (Ag NPs) are formed, with their size being influenced by the redox potential of the reducing agent.

Sanchez-Lopez et al. studied the state of silver in bimetallic Ag-Fe-mordenite catalysts depending on different orders of cation deposition and found a redox interaction between Ag^+^ and Fe^2+^ ions, resulting in the formation of Ag clusters and Ag NPs that show in the DR UV–Vis spectra absorption peak at 370–380 nm for NPs with a size of 1–3 nm and peak above 400 nm for larger Ag NPs. In the TEM images, numerous Ag species with diameters approximately 0.7 nm and larger Ag NPs with sizes in the range of 2–12 nm can be seen [38].

**Figure 3:**
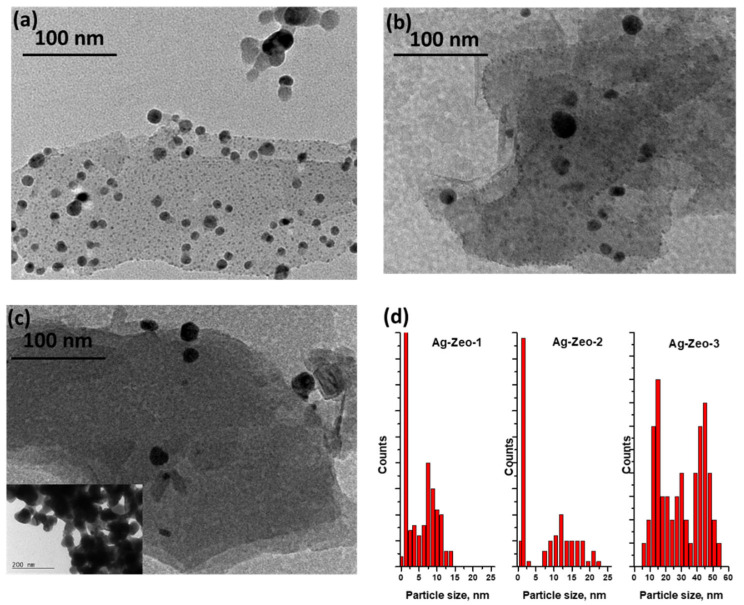
TEM images of (**a**) Ag-Zeo-1, (**b**) Ag-Zeo-2, and (**c**) Ag-Zeo-3 (inlet: image of different area); (**d**) size distribution of the particles.

The TEM images of the samples Ag-Zeo-1, Ag-Zeo-2, and Ag-Zeo-3 and the size distribution of nanoparticles are displayed in Figure 3. As can be seen in Figure 3a, two types of particles exist in the Ag-Zeo-1 composite: ultrafine silver particles with a diameter of 0.5–1 nm and bigger ones with a size in the range of 2–15 nm. One can assume that the predominate particles correlate with the absorption peak at 305 nm and the other fraction gives a shoulder around 380 nm (Figure 2). Many authors also assigned the broad absorption band above 370 nm to plasmon resonance of metallic silver nanoparticles with a size in the range of 1–3 nm [38,42,46,47].

The particles of Ag-Zeo-2 (Figure 3b,d) can be grouped into two ranges, small ones with a size of 0.5–2 nm and spherical particles with a diameter within the range of 7–22 nm. The well-pronounced peak at 385 nm in the DR UV–Vis spectrum (Figure 2) appeared from the latter particles, while a weak peak at 305 nm can be explained by the fraction of ultrafine nanoparticles. Such very small particles were not observed in the TEM image of the Ag-Zeo-3 sample (Figure 3c,d), where the size range is wider, namely from 5 to 55 nm in diameter, which relates to the broad absorption band centered at 450 nm in the DR UV–Vis spectrum (Figure 2). These findings are in accordance with the observation by XRD patterns for the metallic silver phase for sample Ag-Zeo-3 only (Figure 1). If one seeks a deeper insight into the results of the methods, a discrepancy between Ag NP sizes estimated by DR UV–Vis spectroscopy and TEM can be detected, which arises from the different principles of the methods. While TEM provides direct measurements of the real size of nanoparticles, the UV–Vis is sensitive to the optical properties of the particles (depending on the size, shape, surface, and surrounding medium), so the estimated size of the particles is an average value and is indirectly given through the surface plasmon resonance band of Ag NPs.

Overall, the formation of ultrasmall Ag NPs in the Ag-Zeo-1 composite could be attributed to the thermal reduction of silver ions present in the zeolite pores and their movement across the zeolite structure. When a weak reducing agent such as Na_3_Cit is applied in solution, silver ions are desorbed from the zeolite framework and are released in solution, where they are reduced faster, undergoing nucleation and formation of bigger particles (7–22 nm) adsorbed on the surface of the zeolite or on the fracture-type pores. The wider size distribution in the range of 5–55 nm for the Ag-Zeo-3 sample resulted from the rapid reduction of Ag^+^ ions with the strong reducing agent, causing quick movement of the ions into the solution and formation of larger particles that are deposited on the zeolite surface or in the pores between crystal aggregates. Bartolomeu et al. studied the H_2_ reduction of Ag-exchanged zeolites and reported the formation of Ag NPs with a bigger size (about 30 nm) than those found in the calcinated samples (NPs with an average diameter of 3–4 nm) [37].

Reduction of Ag-exchanged zeolite using an aqueous NaBH_4_ solution results in the formation of relatively large Ag NPs, predominantly located on the surface of the zeolite crystals. These nanoparticles are expected to exhibit weaker interactions with the active sites of the zeolite framework. Baltazar et al. found theoretical and experimental evidence for chemisorption of Ag NPs on natural zeolite and described the adsorption and regular distribution of the silver nanoparticles on the outer zeolite surface [48].

**Figure 4:**
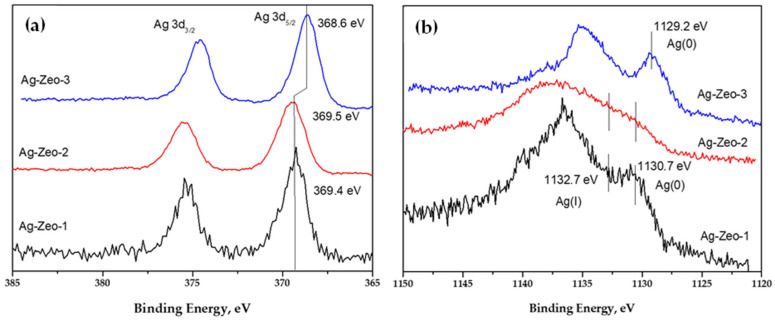
(**a**) Ag 3d XPS and (**b**) Auger spectra of samples Ag-Zeo-1, Ag-Zeo-2, Ag-Zeo-3.

The XPS data (Figure 4) show the oxidation state of silver in the composites. It is clearly seen that the signal for Ag(0) is most intensive for the sample Ag-Zeo-3, showing the predominant portion of metallic silver. This is in accord with the TEM and XRD findings indicating Ag NPs on the zeolite surface. The binding energy for Ag 3d_5/2_ (368.6 eV) for this sample is very close to the literature value for elemental silver (368.3 eV) [49] and for other reported silver-supported zeolites (368.2 eV) [16,50]. This is associated with the formation of larger Ag NPs located on the surface of the substrate, because of the reduction in solution with the strong reducing agent, NaBH_4_.

The Ag 3d_5/2_ peak positions at 369.4 and 369.5 eV for Ag-Zeo-1 and Ag-Zeo-2, respectively, are within the experimental error and could be attributed to the presence of both silver oxidation states Ag(0) and Ag(I). Although the observed binding energy for Ag 3d is higher than that reported for metallic Ag (368.3 and 374.3 eV for Ag3d5/2 and Ag3d3/2) and silver oxides (Ag3d5/2 367.5–368 eV) [49], we consider that the signals of the composites are due to silver in a mixed oxidation state as has also been proposed by other authors [51,52]. Additionally, this is confirmed by the Auger spectra of the samples (Figure 4b). The reference value for the Auger line AgM_4_NN of Ag(0) is 1129 eV [49]. In sample Ag-Zeo-3, the experimental peak is observed at 1129.2 eV, confirming the presence of Ag(0). However, in samples Ag-Zeo-1 and Ag-Zeo-2, the signals are broadened and shifted towards higher values. After deconvolution, it was suggested that these signals may arise from two species—Ag(I) with the binding energy of 1132.7 eV and Ag(0) with the energy of 1130.7 eV [31]. Recently, Sanchez-Lopez et al. also observed a Ag 3d XPS peak above 369 eV and suggested that it related to silver clusters with diameters less than 2 nm [38].

### Antibacterial Activity Against Escherichia coli of Samples Ag-Zeo-1, Ag-Zeo-2, and Ag-Zeo-3

**Figure 5:**
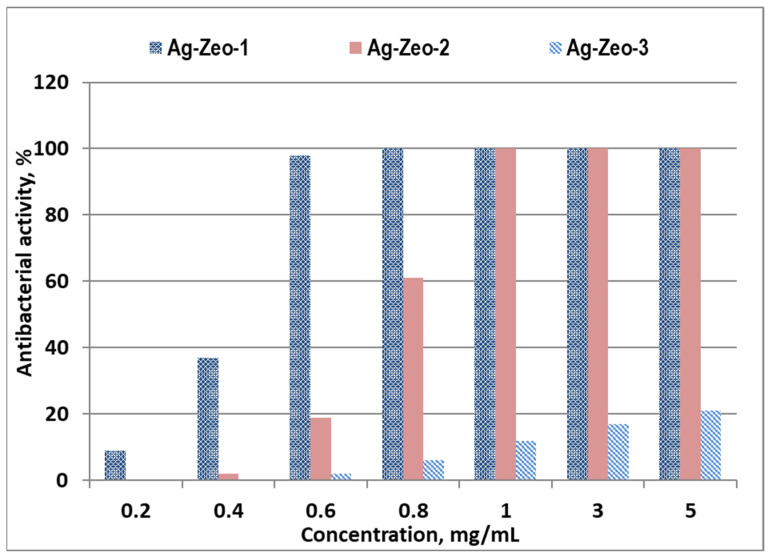
Antibacterial activity against *Escherichia coli* of samples Ag-Zeo-1, Ag-Zeo-2, and Ag-Zeo-3.

The effect of the reduction method of Ag-exchanged zeolite on the antibacterial activity of as-prepared nanocomposites against the Gram-negative bacterium *Escherichia coli* was investigated, and the results are illustrated in Figure 5. As can be seen, the antibacterial activity increases with the increase of nanocomposite concentration. The lowest MIC was found for the sample Ag-Zeo-1 (0.8 mg/mL), followed by Ag-Zeo-2 (1.0 mg/mL). Silver-exchanged zeolite NaY shows an MIC of 0.25 mg/mL against *Escherichia coli* in distilled water, and its antibacterial activity increases with an increase in the concentration of Ag^+^-loaded ions [53]. Thermally treated Ag-zeolite X and Ag-loaded ZSM-5 showed a bactericidal effect on *Escherichia coli* at a concentration of 2.5 mg/mL. The same authors provided evidence that silver ions are the most active species in a series of Ag^+^/Ag NP-containing zeolites [54]. Jiraroj et al. found silver-dose-dependent antibacterial activity of silver-NaA zeolite materials. Ag^+^-NaA composites were more effective against *Escherichia coli* than their counterpart Ag NPs-NaA composites, prepared by the reduction of Ag^+^-NaA with NaBH_4_ [55]. The weakest antibacterial activity among the samples studied was observed for Ag-Zeo-3. This could be explained by the larger Ag NP size in comparison with the dimensions of silver species in the samples Ag-Zeo-1 and Ag-Zeo-2. Therefore, the antibacterial performance of the three samples can be attributed to the NP size, type of silver species, and their distribution within the zeolite support. It is worth mentioning that antibacterial activity for a control sample of natural zeolite was not found, indicating the crucial role of silver in the nanocomposites’ properties. In contrast, slight stimulation of the bacterial growth was observed over natural zeolite [30]; similar results for clinoptilolite were reported by other authors [34].

Generally, the mechanisms of antibacterial activity of Ag-containing materials are still not very well understood, but experimental investigations reveal that factors such as size, morphology, surface area, and stability of Ag NPs can affect the effectiveness of Ag treatment. The proposed Ag NP uptake in cells involves various processes. On one hand, nanoparticles can cross the cell membrane by interaction with membrane proteins, followed by intracellular generation of reactive oxygen species that lead to cell damage and cell death. On the other hand, Ag NPs may release Ag^+^ ions outside the cell by dissolution and oxidation, and then Ag^+^ ions can easily penetrate the cell and can react with sulfur- and nitrogen-containing molecules in the membrane, cytoplasm, and mitochondria, causing DNA and protein changes, enzyme inactivation, and mitochondrial dysfunction, leading to apoptosis [35,56].

As shown above, the reduction of Ag-exchanged zeolite by NaBH_4_ gives NPs with a diameter of 5–55 nm located on the zeolite surface. Such size and aggregation of Ag NPs inhibit the oxidation to Ag^+^ ions and the possibility for physical contact with the bacterial membrane surface, thus reducing the antibacterial potential of this sample. In contrast, the Ag-Zeo-1 sample contains numerous ultrasmall Ag species uniformly dispersed within the zeolite framework, along with 2–15 nm-sized Ag NPs and residual immobilized Ag^+^ ions. Both the unreduced Ag^+^ ions and those obtained through gradual dissolution of Ag NPs can be released in biological environments, enhancing silver bioavailability in both extracellular and intracellular media, and promoting more effective interactions with bacterial cells.

According to previous studies, NPs with a size less than 10 nm can penetrate the bacterial membrane and cause oxidative stress, DNA damage, cellular degradation, and subsequent bacteria death [2,56]. It was found that 10 nm-sized Ag NPs showed greater activity against *Escherichia coli* than particles over 50 nm in size [57], which related to the fact that smaller Ag NPs release more Ag^+^ ions and show higher Ag bioavailability than that of 50 nm-sized NPs. We can propose that the higher antibacterial activity of Ag-Zeo-1 is partially due to the available Ag^+^ ions in the zeolite support, and silver clusters and nanoparticles with diameters up to 15 nm that are dissolved in the biological media. For the Ag-Zeo-2 sample, the ratio between Ag species within the composite is altered, resulting from a higher degree of Ag(I) reduction, nucleation, and particle growth, reaching a maximum size of Ag NPs up to 22 nm. This structural change of the composite is associated with a decline of biocidal activity. Thus, the current study confirmed the hypothesis that Ag^+^ ion release in solution is an essential step in antibacterial action, which occurs via interaction and damage of the bacterial cell wall and its internal components [54,55].

Missengue et al. investigated the leaching of silver from Ag-zeolite samples, containing Ag^+^ ions and Ag NPs formed by chemical reduction with NaBH_4_ solution. They reported that the leaching rate and antibacterial activity decreased after the reduction of Ag^+^ ions regardless of Ag^+^ loading on the support [58]. The antibacterial and antifungal properties of Ag/zeolite Y, prepared by the reduction of Ag^+^-exchanged zeolite, were also reported [33]. Top et al. proved antibacterial activity of Ag^+^-exchanged clinoptilolite [59]. Furthermore, other authors demonstrated the advantages of Ag^+^-loaded clinoptilolite for water disinfection and heavy metal removal [60]. By using the electrochemical method, Inoue and Kanzaki observed that the antibacterial activity of Ag-zeolite drastically decreases when Ag^+^ ions are reduced to metallic silver and explained the role of Ag(I) in DNA and cell membrane damage and enzyme inhibition [61,62].

## 4. Conclusions

In conclusion, we demonstrate that the composition, surface chemistry, and Ag species within the composites significantly influence their antibacterial properties, which vary depending on the preparation method used. Thermal reduction of solid Ag-exchanged zeolite resulted in the formation of Ag clusters and Ag nanoparticles (NPs) within the zeolite structure (Ag-Zeo-1), whereas Ag^+^ ions were only partially reduced under ambient air conditions. Deeper conversion of Ag^+^ to Ag NPs was achieved through reduction with sodium citrate (Na_3_Cit) in aqueous solution at room temperature, which was catalyzed by triethanolamine (Ag-Zeo-2). In contrast, using a strong reducing agent such as sodium borohydride (NaBH_4_) yielded polydisperse and larger Ag NPs (up to 55 nm) that aggregated and adsorbed onto the zeolite surface (Ag-Zeo-3). The DR UV–Vis spectroscopy revealed distinct absorption bands associated with different silver species: at 222 nm for Ag^+^ ions, at 305 nm for silver clusters, and at 380 nm for Ag NPs in the sample Ag-Zeo-1, while more intense bands at 385 nm and 450 nm characterized Ag NPs in the samples Ag-Zeo-2 and Ag-Zeo-3. These spectral observations were consistent with TEM analysis, which confirmed the coexistence of ultrafine particles and Ag NPs with a size of 0.5–1 nm and 2–15 nm in Ag-Zeo-1, slightly larger particles with a diameter of 0.5–2 nm and 7–22 nm in Ag-Zeo-2, and the broader size distribution of Ag NPs, 5–55 nm, in Ag-Zeo-3. The antibacterial properties of nanocomposites were assessed by the determination of MIC concentrations against *Escherichia coli* that were found to be 0.8 mg/mL for Ag-Zeo-1 and 1.0 mg/mL for Ag-Zeo-2. The highest antibacterial activity was observed for thermally reduced Ag-zeolite, which contained both Ag^+^ ions and uniformly distributed Ag NPs throughout the zeolite matrix. This configuration facilitated the slow release of Ag^+^ ions and provided the most effective bacterial inhibition. Therefore, thermal reduction of Ag-exchanged zeolite was evaluated as a promising approach for preparing nanocomposites with enhanced antibacterial properties.

## Figures and Tables

**Table 1:** Characteristic bands present in the DR UV–Vis spectra of the nanocomposites.

	Ag-Zeo-1	Ag-Zeo-2	Ag-Zeo-3	Assignment
UV–Vis	222 (s)	222 (sh)	222 (w)	Ag^+^ ions in zeolite
band	257 (m)	257 (w)	257 (w)	Zeolite network
(nm)	305 (m)	305 (w)	305 (sh)	Ag clusters
	380 (sh)	385 (s, br)		Ag NPs 1–3 nm
			450 (s, br)	Ag NPs > 15 nm

(s) strong; (m) medium; (w) weak; (sh) shoulder; (br) broad.

## Data Availability

The original contributions presented in the study are included in the article, and further inquiries can be directed to the corresponding author.
